# The Role of Methylacrylylated Hyaluronic Acid Hydrogels in Promoting Skin Wound Healing

**DOI:** 10.5812/ijpr-169881

**Published:** 2026-05-04

**Authors:** Chunfang Zheng, Waifung Mai, Liqi Zou, Lianying Li, Bingwei Sun

**Affiliations:** 1 Department of Burn and Plastic Surgery, Affiliated Suzhou Hospital of Nanjing Medical University, Suzhou, China; 2 Department of Dermatology, Nanjing University Drum Tower Hospital, Nanjing, China

**Keywords:** Wound healing, Macrophage, Fibroblasts, Methylacrylylated hyaluronic acid, Single-cell transcriptome

## Abstract

**Background:**

Hyaluronic acid, as a natural extracellular matrix component, possesses excellent biocompatibility and moisturizing properties, and demonstrates unique advantages in wound repair.

**Objectives:**

To explore the role of hyaluronic acid methacryloyl (HAMA) hydrogels in skin wound healing and analyze the characteristics of the local microenvironment of wounds.

**Methods:**

Mice divided into a control group (n = 6) and a HAMA group (n = 6) at random were used to create a model of total cortical resection. 100μL of HAMA hydrogel was applied to the wound surface in the HAMA group, while 100 μL of lithium phenyl-2,4,6-trimethylbenzoyl hypophosphonate (LAP) was applied to the wound surface in the control group. Both were irradiated with an ultraviolet lamp for 20 seconds, and on days 0, 3, 7, 10, and 14, the residual wounds were measured. The effect of the HAMA hydrogel on wound healing was analyzed by measuring the remaining wound area and performing hematoxylin-eosin (H&E) staining. The cellular characteristic spectrum of the local skin of the wound on the 14th day was analyzed via single-cell sequencing technology, and the degree of type I and type III collagen expression, F4/80, CD206 and CD86 in the local wound were detected via immunohistofluorescence technology. The mRNA expression levels of Arg1, Nos2, Itgam and Itgb2 in the RAW264.7 mouse macrophage line coincubated with the HAMA hydrogel for 24 hours were detected by RT-qPCR. Cluster analysis of fibroblasts and macrophages in the local skin of the wounds on the 14th day in mice was conducted via the Seurat software package, and the communication status between fibroblasts and macrophages was analyzed via the CellChat software package.

**Results:**

The HAMA group's skin wounds healed considerably more quickly than the control group's did. While the wounds in the control group had not yet fully healed, those in the HAMA group had by the fourteenth day. Single-cell sequencing analysis revealed that the proportion of fibroblast subsets with high expression of Col3a1 in the HAMA group (90.2%) was greater than that in the control group (79.8%), whereas the proportion of fibroblast subsets with high expression of Colla1 (5.7%) was lower than that in the control group (15.9%). The results of the immunofluorescence analysis confirmed that the local type III collagen level in the wounds of the HAMA group was greater than that in the wounds of the control group (P = 0.035), whereas the type I collagen level was lower than that in the wounds of the control group (P = 0.044). There was no significant difference in the proportion of local macrophages on the wound surface between the HAMA group mice and the control group mice. However, both the single-cell sequencing analysis results and the in vitro treatment of Raw264.7 macrophages with the HAMA hydrogel revealed increased expression of Arg1 (P < 0.001) and decreased expression of Nos2 (P < 0.001). Moreover, in the HAMA group, macrophages at the wound site expressed higher levels of CD206 (P = 0.042) and lower levels of CD86 (P = 0.011).

**Conclusions:**

Treatment of the microenvironment with the HAMA hydrogel is conducive to the healing of skin wounds, and more anti-inflammatory macrophages and fibroblasts that secrete type III collagen accumulate locally in the wound healing tissue.

## 1. Introduction

The first line of defense for the immune system is the skin, the biggest organ in the human body (1). It is crucial for maintaining the homeostasis of the body. Delayed wound healing caused by external injuries, infections and chronic inflammation seriously affects the health of the body (2- 4). Therefore, constantly improving the effectiveness of wound healing is an important issue faced in clinical practice (5). There are four primary phases to the ongoing, intricate process of skin wound healing: the hemostatic stage, the inflammatory stage, the regenerative stage and the remodeling stage. During the healing process, various cells and the molecules they secrete jointly complete this delicate biological process (6- 8). During the inflammatory period, many inflammatory cells can be recruited locally at the wound site and released through calcium ions. Mechanisms such as release and increased reactive oxygen species are used to eliminate pathogenic bacteria and damaged cells, among which neutrophils and macrophages are particularly crucial (9- 11). During the regenerative and remodeling phases, macrophages can also recruit many fibroblasts to the wound site. Fibroblasts secrete collagen, causing continuous accumulation of collagen fibers, thereby completing the repair and remodeling of the wound area (11). Therefore, analyzing the characteristics of the tissue microenvironment during the wound healing process and clarifying the time phases and functions of various cells at play are highly important for promoting wound healing in clinical practice.

Nowadays, a lot of clinical practice uses wound dressings to help skin wounds heal (12). In order to stop external infections, wound dressings can temporarily cover the wound and act as a barrier, which is conducive to the remodeling of skin cells (13- 15). Hydrogels, as high-molecular-weight substances with water solubility or hydrophilicity, mainly form gels with three-dimensional network structures through chemical or physical crosslinking. Its polymer sources can be natural hydrophilic polymers, including polysaccharides (starch, cellulose, alginic acid, chitosan, etc.), polypeptides (collagen, polyL-lysine, polyL-glutamic acid, etc.), or synthetic hydrophilic polymers (such as alcohols, acrylic acids and their derivatives, etc.). hyaluronic acid (HA), a major component of the extracellular matrix, is widely distributed in the skin, connective tissue and joints of the body and has multiple functions (16). It also features degradability, nonimmunity, viscoelasticity, targeting and good biocompatibility. It has already demonstrated broad application prospects in the field of biomedicine (17). However, the half-life of HA is short, and its adhesion is poor. This problem can be solved by chemically modifying or cross-linking the carboxyl or hydroxyl ends of HA. At present, HA derivatives have made progress in preventing postoperative adhesions, treating osteoarthritis and constructing scaffold materials for tissue engineering. hyaluronic acid methacryloyl (HAMA) is a chemically modified HA derivative that is prepared by adding methacrylate anhydride to the carboxyl end of HA (18). The methyl propylene group on the HAMA molecular chain endows it with photocuring properties, enabling it to cross-link through photopolymerization to form hydrogels. After curing, HA molecules exhibit excellent mechanical strength, adhesion and biocompatibility. Loading active ingredients onto HAMA hydrogels can promote wound healing (19- 21). However, there have been no reports on how HAMA hydrogels regulate the microenvironmental characteristics of wound healing.

The current research on the application of methylacrylated hyaluronic acid (HAMA) hydrogels in skin wound healing mainly focuses on their physical barrier and moisturizing functions, while systematic studies on their immunomodulatory effects are still insufficient. Especially in the wound microenvironment, how HAMA precisely regulates the phenotypic transformation of macrophages and how this regulation further affects the interaction between macrophages and fibroblasts, thereby promoting tissue repair, has not been fully elucidated. The existing literature mostly remains at the level of phenomenon observation and lacks in-depth exploration of the "macrophage-fibroblast" dialogue network, which limits our overall understanding of the healing mechanism of HAMA hydrogels. This study aims to fill this research gap by systematically revealing the regulatory effect of HAMA hydrogels on macrophage polarization and its interaction mechanism with fibroblasts through single-cell sequencing and other technologies, providing a theoretical basis for the development of intelligent wound repair materials with precise immunomodulatory functions, and has significant scientific value and clinical translation potential.

## 2. Methods

### 2.1. Cell Culture

The Raw264.7 mouse macrophage cell line provided by the Cell Bank of the Chinese Academy of Sciences was used for the experiment. During cell recovery, the frozen tubes were taken out from the liquid nitrogen tank and immediately placed in a 37°C water bath for rapid thawing. Then, they were transferred to a complete medium containing 10% fetal bovine serum, and centrifuged at 1000 rpm for 5 minutes. The supernatant was discarded and the cells were re-suspended in the high-glucose DMEM medium (HyClone, USA) supplemented with 5% fetal bovine serum (Gibco, USA), 50 U/mL penicillin/streptomycin (Gibco, USA), 2 mmol/L L-glutamine (Gibco, USA), and 20 mmol/L HEPES (Gibco, USA). The cells were cultured in a 37°C, 5% CO2, 95% relative humidity incubator. When the cells reached 80%-90% confluence, they were digested and passaged using 0.25% trypsin-EDTA, with a passage ratio of 1:3 to 1:5. The cells were seeded in the corresponding culture plates 24 hours before the experiment to ensure they were in the logarithmic growth phase and the cell viability was detected by trypan blue staining, ensuring that the proportion of viable cells was above 95%. All the culture media were freshly prepared and stored at 4°C. They were preheated to 37°C before use.

### 2.2. Experimental Animals

Female SPF-grade C57BL/6 mice were used as experimental animals. All mice were purchased from Lingchang Company, with an initial weight of 18 - 22g and an age of 6 - 8 weeks. After arriving at the laboratory, they were given a 7-day adaptation period. During this period, they were housed in standard polycarbonate cages with 5 mice per cage, and the bedding was changed twice a week. The feed was SPF-grade mouse maintenance feed, and the water was sterilized pressurized tap water, freely consumed through drinking bottles. The environmental temperature was maintained at 22 ± 2°C, the relative humidity was 55%-65%, and the light cycle was 12 hours of alternating light and dark, with an illumination intensity of approximately 300 lux. All experimental procedures were in accordance with the "Guidelines for the Welfare and Ethics of Laboratory Animals" and were approved by the Animal Ethics Committee (Approval Number: HKYS-2025-A0185). 24 hours before the experiment, all mice were fasted but not deprived of water to reduce surgical stress responses. The animals were randomly divided into the control group and the experimental group, with 6 mice in each group, ensuring no statistical difference between the groups. During the experiment, the health status of the animals was closely monitored, and any abnormalities were immediately addressed.

Isoflurane was used as the anesthetic agent and administered through an inhalation anesthesia system. During the induction stage, a concentration of 3.5% isoflurane was used, and during the maintenance stage, the concentration was adjusted to 1.5 - 2.0%. The oxygen flow rate was set at 1.5L/min. The depth of anesthesia was determined by evaluating corneal reflex, plantar reflex, and muscle tone, ensuring that the animals were in an appropriate anesthetic state during the surgery. For postoperative pain management, buprenorphine was used as the analgesic drug, with a dose of 0.1mg/kg body weight, administered subcutaneously. The drug was given once 30 minutes before the surgery and once 12 hours after the surgery.

### 2.3. Preparation and Characterization of the HAMA Hydrogel

The hydrogel was prepared using methylacrylated hyaluronic acid (HAMA). Firstly, the HAMA powder was accurately weighed and dissolved in phosphate-buffered saline under sterile conditions to form a 1% (w/v) solution. The solution was stirred magnetically until complete dissolution to obtain a homogeneous solution. The photoinitiator system was prepared by mixing 2-hydroxy-2-methylbenzophenone with lithium phosphate in a 1:2 molar ratio. It was stored in the dark at 4°C. The HAMA solution was mixed with the photoinitiator system at a volume ratio of 9:1, thoroughly mixed, and transferred to a specially designed mold. It was then placed under a 365nm ultraviolet lamp at a distance of 15cm, irradiated at a power of 5mW/cm² for 10 minutes to complete the crosslinking reaction. After the preparation was completed, the mechanical properties of the hydrogel were evaluated by rheological testing, the microstructure was observed using a scanning electron microscope, the chemical crosslinking degree was confirmed by Fourier transform infrared spectroscopy, and the swelling rate and degradation characteristics were determined. The application frequency of HAMA hydrogel was once every 48 hours. All operations were carried out under sterile conditions to ensure the biocompatibility and application safety of the hydrogel.

The methacrylated hyaluronic acid (HAMA) was synthesized by a one-step method. Sodium hyaluronic acid (with a molecular weight of 100 kDa) was reacted with methacrylic acid anhydride under alkaline conditions. The reaction temperature was controlled at 25°C, and the pH was maintained at 8.5. The reaction time was 4 hours. After purification by dialysis, the degree of methacrylation was determined by nuclear magnetic resonance hydrogen spectroscopy (^1H NMR). The results showed that each disaccharide unit contained an average of 1.8 methacryloyl groups. The rheological test was conducted using a rotational rheometer, and the storage modulus (G') and loss modulus (G'') of the hydrogel were measured at 37°C. The frequency scanning range was 0.1 - 10 Hz. The swelling rate was determined by immersing the dry weight of the hydrogel (W_d) in PBS, weighing regularly until reaching the equilibrium swelling state (W_s), and calculating the swelling rate SR = W_s/W_d. The degradation experiment was carried out in PBS at 37°C and pH = 7.4. Samples were taken regularly to determine the residual mass, and the degradation curve of the hydrogel's in vitro degradation kinetics was plotted to evaluate the hydrogel's in vitro degradation dynamics characteristics.

### 2.4. Establishment of a Wound Healing Model of Total Cortical Resection in Mice

The control group (n=6 ) and the HAMA group (n=6 ) were randomly selected. After the mice were anesthetized, the fur on their backs was shaved. A 10 mm circular perforator was used to circle the shape and size of the skin wound on the mouse's back, and then the entire layer structure of the circled skin was cut off. On the wound surface, 100 μL of HAMA hydrogel was applied to the HAMA group, while 100 μL of LAP was applied to the control group. An ultraviolet lamp was used to irradiate both for twenty seconds. On the 0th, 3rd, 7th, 10th, and 14th days, the remaining wounds were measured. The current wound area divided by the initial wound area times 100% is the remaining wound area (%). On the 14th day, all the mice were sacrificed. Skin tissues with a diameter of 12 mm around the center of the wound were cut for subsequent single-cell transcriptome analysis and pathological tissue detection.

### 2.5. Hematoxylin-eosin (H&E) Staining

Hematoxylin-eosin (H&E) staining was used for histological analysis of the skin tissue. After the tissue samples were fixed with 4% paraformaldehyde for 24 hours, they were dehydrated with gradient ethanol (70% → 80% → 95% → 100% for each 1 hour), and then treated with xylene for transparency (twice, each 30 minutes). After being embedded in paraffin (twice, each 1 hour), the tissue was sectioned into 4 μm thick consecutive sections using a Leica RM2235 microtome. The sections were then baked in a 60°C oven for 30 minutes, followed by dewaxing and gradient hydration. The staining process strictly followed the standard protocol: hematoxylin staining for 5 minutes, 1% hydrochloric acid alcohol differentiation for 15 seconds, ammonia re-blotting for 30 seconds, and water rinsing for 5 minutes; eosin staining for 2 minutes, gradient ethanol dehydration (85% → 95% → 100%, each 1 minute), xylene transparency (twice, each 2 minutes). Finally, the sections were sealed with neutral gum, and the whole section was scanned using a Seville digital slide scanner (Wuhan, China) at a resolution of 40x to ensure clear tissue structure, providing a reliable basis for the histological evaluation of skin wound healing.

### 2.6. Fluorescence Staining of Immunotissue

Antigen remediation was performed on the sections via antigen remediation buffer. To decolorize the sections, they were submerged in phosphate-buffered saline (PBS) after naturally cooling. Using a closed histochemical pen, circles were drawn around the tissue. After being submerged in a 3% hydrogen peroxide solution, the sections were left to incubate for twenty-five minutes at room temperature in the dark. After washing and drying, 3% fetal bovine serum (FCS) was added, and the samples were blocked for 30 minutes. The samples were incubated at 4°C for the entire night after the addition of the primary antibody. Once the samples were cleaned and dried, the matching secondary antibody that was labeled with the fluorescent was applied, and the samples were incubated for 50 minutes at room temperature. After cleaning, the tissue sections were placed in antigen retrieval buffer and heated in a microwave oven. After the samples were washed and dried, the corresponding fluorescent secondary antibody was added, and the samples were incubated at room temperature in the dark for 50 minutes. After the samples were washed and dried, DAPI staining solution was added to the cell nucleus for counterstaining, and the samples were incubated at room temperature in the dark for 10 minutes. After rewashing and spin-drying, an autofluorescence quencher was added to the circle. Anti-fluorescence quenching mounting medium was used for mounting, and images were collected via a digital section scanner (Seville, Wuhan, China).

### 2.7. Single-Cell Sequencing

The cell heterogeneity during the wound healing process was analyzed using single-cell sequencing technology. Skin tissue was collected within a 12mm diameter range around the wound center. The epidermis layer was precisely separated using an epidermal separation kit and the resulting epidermal layer was filtered through a 70μm cell filter to obtain a single-cell suspension. The dermal tissue was cut into 1mm³ pieces and added to 0.1% type I collagenase and 0.25% trypsin. The mixture was digested in a 37℃ water bath for 45 minutes, with gentle shaking every 15 minutes. The digested tissue suspension was filtered through a 100μm filter, centrifuged at 300×g for 5 minutes, the supernatant was discarded, and the tissue was treated with red blood cell lysis buffer for 5 minutes before centrifugation and resuspension in DMEM medium containing 10% FBS. It was stored at 4℃ for future use. The construction of single-cell sequencing libraries and the original data analysis were completed by Beijing Xun Yin Biotechnology Co., Ltd. High-throughput sequencing was performed using the 10× Genomics platform to ensure the acquisition of high-quality sparse matrix data, laying the foundation for subsequent cell subpopulation identification and functional analysis.

After cell dissociation, we used the trypan blue staining method to assess the cell survival rate, ensuring that the cell survival rate of all samples was above 90%, with a specific range of 92% to 95%. This ensured the reliability of the subsequent sequencing data. For each sample, we captured approximately 8,000 to 10,000 cells. This quantity was determined based on previous pre-experiments and was sufficient to fully cover the main cell subpopulations in the wound microenvironment. Sequencing was performed using the 10x Genomics Chromium X platform, using the Single Cell 3' Reagent Kit v3 for library construction. This platform is one of the mainstream technologies in the field of single-cell sequencing. In terms of sequencing depth, we obtained an average of approximately 50,000 UMI and 5,000 detected genes per cell, with a median gene count of 4,800, and the sequencing saturation reached over 85%. These indicators all met the standards for high-quality single-cell sequencing. In terms of quality control, we strictly filtered out low-quality cells (with gene numbers < 500 or > 6,000, and UMI numbers < 2,000), and cells with mitochondrial gene proportions exceeding 20% were also excluded to ensure the reliability of the data.

### 2.8. Raw264.7 Cells Cocultured with HAMA Hydrogels in vitro

The effect of HAMA hydrogel on Raw264.7 macrophages was evaluated using an in vitro co-culture system. Firstly, 5 mg of photoinitiator LAP (Lithium phenyl-2,4,6-trimethylbenzoylphosphinate) was accurately weighed and dissolved in 1 mL of sterile double-distilled water. Then, 10 mg of HAMA powder was added and magnetic stirring was performed at 4°C until complete dissolution to form a homogeneous solution. The above solution was evenly spread at a volume of 200 μL per well on the bottom of a 6-well plate and placed under a 365 nm ultraviolet lamp at a distance of 10 cm for 5 minutes to complete the cross-linking reaction. After cross-linking, the solution was gently washed twice with PBS to remove unreacted substances. Meanwhile, Raw264.7 cells in the logarithmic growth phase were digested with trypsin, centrifuged, and resuspended in DMEM medium containing 10% FBS, adjusting the cell concentration to 1 × 106 cells/mL. 1 mL of the cell suspension was carefully dropped onto the surface of the hydrogel to ensure uniform distribution of the cells. The 6-well plate was placed in a culture box at 37°C, 5% CO2, and saturated humidity for 24 hours, with the untreated wells serving as the blank control group. After the culture, the cells were collected for subsequent analysis..

### 2.9. Reverse Transcription Real-time Fluorescence Quantitative PCR

The expression level of the target gene was detected by reverse transcription real-time fluorescence quantitative PCR technology. After cell collection, total RNA was extracted using TRIzol reagent according to the standard procedure. The RNA purity (A260/A280 ratio between 1.8 and 2.0) and concentration were detected using a NanoDrop spectrophotometer. 500 ng of high-quality RNA was taken and reverse transcribed using the Takara reverse transcription kit in a 20 μL reaction system containing 5× PrimeScript Buffer 2 μL, PrimeScript RT Enzyme Mix I 1 μL, Oligo dT Primer 1 μL, Random 6 mers 1 μL and total RNA. The reaction conditions were 37°C for 15 minutes, 85°C for 5 seconds, and then stored at 4°C. Subsequently, a qPCR reaction was performed using the TB Green® Premix Ex TaqTM Ⅱ reagent kit. The 20 μL reaction system contained TB Green Premix Ex Taq II 10 μL, upstream and downstream primers each 0.8 μL, cDNA template 2 μL and RNase Free dH2O 6.4 μL. The reaction conditions were 95°C for 30 seconds pre-denaturation, 40 cycles (95°C 5 seconds, 60°C 30 seconds, 72°C 30 seconds) for the PCR. Gapdh was used as the internal reference gene, and the mRNA relative expression levels of Arg1, Nos2, Itgam and Itgb2 were calculated using the 2−ΔΔCt method. Three technical replicates were set for each sample, and the experiment was repeated three times to ensure the reliability of the results. The primer sequences were designed and synthesized by Shanghai Biotechnology Engineering Co., Ltd., and were verified to have good specificity.

The antigen retrieval buffer is a citrate buffer (pH 6.0), purchased from Beijing Solibo Technology Co., Ltd.; the tissue fixation solution is a 4% paraformaldehyde solution, provided by Beijing Dingguochangsheng Biotechnology Co., Ltd.; the bluing solution is a 1% lithium carbonate aqueous solution, prepared by the laboratory itself; the primary antibodies include anti-CD68, anti-iNOS and anti-CD163 antibodies, all purchased from Abcam Company; the secondary antibody is HRP-labeled rabbit anti-mouse IgG, purchased from Cell Signaling Technology; the antigen retrieval buffer is EDTA buffer (pH 8.0), purchased from Beijing Baolabole Technology Co., Ltd.

The anti-CD68 antibody was purchased from Abcam (catalog number ab91151), the anti-iNOS antibody from Cell Signaling Technology (catalog number C130302), the anti-CD163 antibody from R&D Systems (catalog number MAB15673), and the anti-β-actin antibody from Sigma (catalog number A5441).

The sequences of the qPCR primers were designed and synthesized by Shanghai Biotechnology Engineering Co., Ltd.

The specific sequences are as follows:

The forward primer for the Arg1 gene is 5'-CCCTGAAGGAGAAGCTGTCG-3', and the reverse primer is 5'-TGGTCACAGCCAGGTAGAGC-3';

the forward primer for the Nos2 gene is 5'-GCTGTGCTGTACCTGAACCT-3', and the reverse primer is 5'-CCTCTGGTAGGAGCGGTCTT-3';

the forward primer for the Itgam gene is 5'-TGGAGGAGATGGAAGACCTG-3', and the reverse primer is 5'-CACAGCCACAGTCACAGAGC-3';

the forward primer for the Itgb2 gene is 5'-GCTGGAGGAGATGGAAGACC-3', and the reverse primer is 5'-CACAGCCACAGTCACAGAGC-3';

the forward primer for the Gapdh gene is 5'-AGGTCGGTGTGAACGGATTTG-3', and the reverse primer is 5'-TGTAGACCATGTAGTTGAGGTCA-3'.

All primers were verified for specificity by BLAST, and the amplification efficiency was between 95% and 105%.

### 2.10. Statistical Analysis

All experimental data were statistically analyzed using GraphPad Prism 9.0 software to ensure the accuracy and reliability of data processing. For comparisons between two groups, an independent sample t-test was used to analyze the significance of differences. This test first confirmed the normal distribution of the data through the Shapiro-Wilk test, and then evaluated the homogeneity of variance through the Levene test. Once the conditions were met, the t-test was conducted. All experiments were set with at least 3 biological replicates, and each replicate contained 3 technical replicates to ensure the stability and reproducibility of the results. The statistical significance level was set at P < 0.05. P < 0.01 was marked as a highly significant difference, and P < 0.001 was marked as a highly significant difference. All data were presented as the mean ± standard deviation (±SD) and were visually displayed as differences between groups through bar charts.

## 3. Results

### 3.1. Role of HAMA Hydrogels in Wound Healing

**Figure 1.**
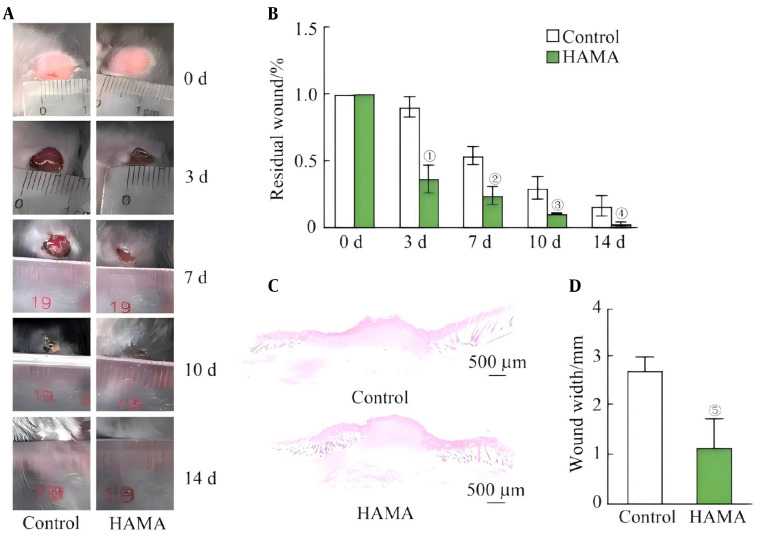
A, Residual wound area; B, statistical graph of the residual wound area (n = 6); C, H&E staining of the wound areas on day 14; D, statistical analysis of the wound width data (n = 6). ①P = 0.002, ②P = 0.006, ③P = 0.017, ④P = 0.039, ⑤P = 0.013, compared with the control group.

After adding the blue light reactant LAP, under irradiation with an ultraviolet lamp, the crosslinking and curing of HAMA results in the formation of a hydrogel. The remaining wound area in the HAMA group was substantially less than that in the control group on days three, seven, ten, and fourteen (Figure 1A and B). By day 14, the mice in the HAMA group had fully recovered from their wounds, while the animals in the control group had not. Additionally, the H&E staining results on day 14 showed that the HAMA group's wounds were noticeably narrower than those in the control group (Figure 1C and D).

### 3.2. Local Cell Characteristic Spectrum of the Wound Surface

**Figure 2.**
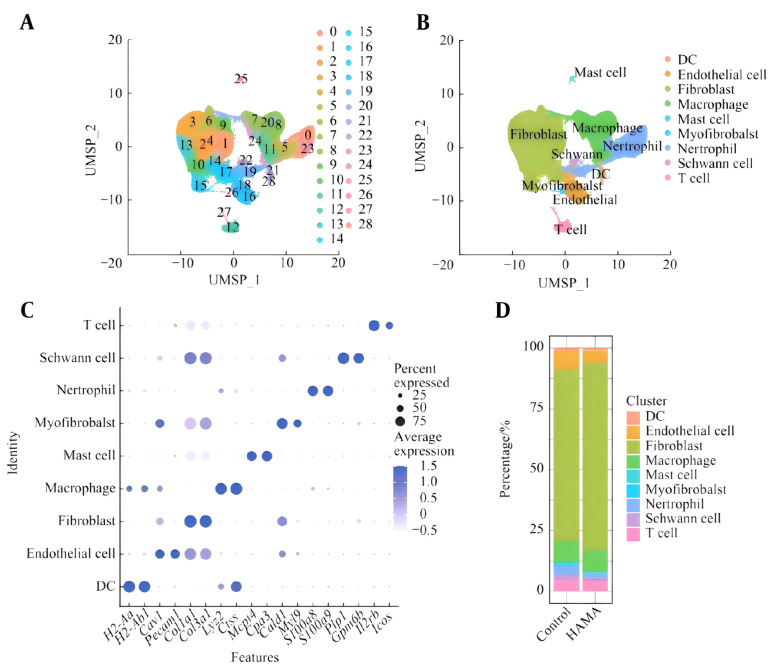
A, Schematic diagram of cell clusters; B, cell types identified at the wound site; C, marker genes for various cell types; D, percentages of various cell types.

After the cells were filtered and subjected to quality control, they were divided into 28 clusters through dimensionality reduction clustering (Figure 2A). The 28 cell clusters were divided into 9 types of cells through characteristic genes (Figure 2B), namely, dendritic cells (Dendritic cells, H2-Aa and H2-Ab1), endothelial cells (Cav1 and Pecam1), fibroblasts (Col1a1 and Col3a1), macrophages (Lyz2 and Ctss), fat cells (Mcpt4 and Cpa3), myofibroblasts (Cald1 and Myl9), neutrophils (S100a8 and S100a9), Schwann cells (Plp1 and Gpm6b), and T cells (Icos and Il2rb) (Figure 2C). On the 14th day, the proportions of the different cell types in the wound tissues of the mice were compared. The proportion of fibroblasts in the wound tissue of the HAMA group (77.2%) was significantly greater than that in the control group (70.7%), whereas the proportion of macrophages in both groups was 8.7% (Figure 2D).

### 3.3. Characteristic Spectrum of Local Fibroblasts on the Wound Surface

**Figure 3.**
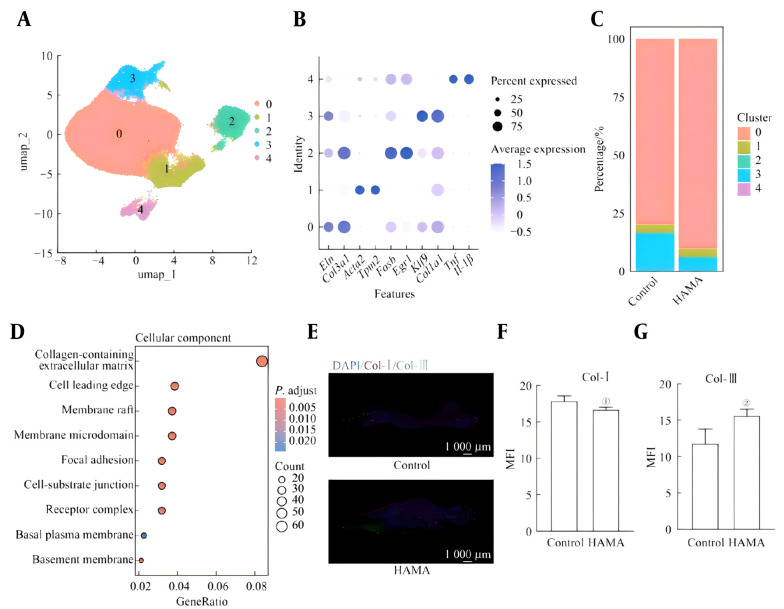
A, Subsets of fibroblasts; B, marker genes of fibroblast subsets; C, percentages of fibroblast subsets; D, GO enrichment analysis of DEGs between fibroblasts; E, immunofluorescence of collagen in the wounds of the mice. F/G. Mean fluorescence intensity of F, type Ⅰ collagen and G, type Ⅲ collagen in the wound area of the mice (n = 6). ①P = 0.044, ②P = 0.035, compared with the control group. MFI-mean fluorescence intensity.

After dimensionality reduction clustering of the fibroblasts, five cell clusters (Clusters 0 to 4) were obtained, as shown in Figure 3A. Among them, specific macrophage subpopulations Cluster 0 expresses Eln and Col3a1, Cluster 1 expresses Acta2 and Tpm2, Cluster 2 expresses Fosb and Egr1, and Cluster 3 expresses Col1a1 and Klf9. Cluster 4 expressed Tnf and Il-1β (Figure 3B). The proportion of fibroblast subsets with high expression of Col3a1 in the HAMA group (90.2%) was greater than that in the control group (79.8%), whereas the proportion of fibroblast subsets with high expression of Col1a1 (5.7%) was lower than that in the control group (15.9%) (Figure 3C). GO enrichment analysis was conducted on DEGs. The "collagen-containing extracellular matrix" pathway exhibited the greatest difference between the two groups (Figure 3D). Type I and type III collagen were subjected to immunofluorescence labeling at the mice's wound site on the fourteenth day. The HAMA group expressed a greater level of type III collagen, while the expression level of type I collagen was lower (Figure 3E-G).

### 3.4. Characteristic Spectrum of Macrophages in the Local Wound Area

**Figure 4.**
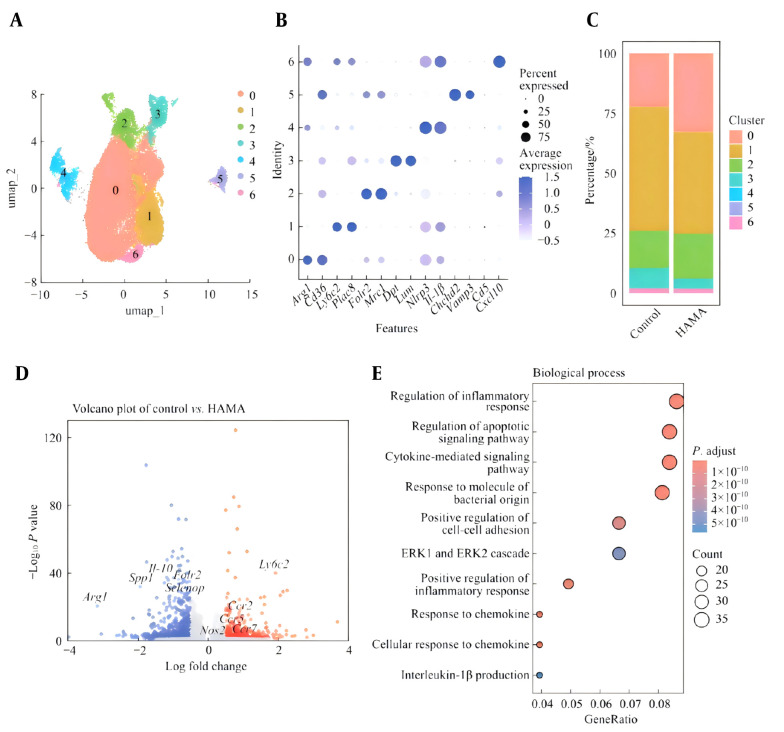
A, Subsets of macrophages; B, marker genes of macrophage subsets; C, percentages of macrophage subsets; D, volcano plot of DEGs in all the macrophages; E, GO enrichment analysis of DEGs.

The functional characteristics of local macrophages in the wounds of the two groups of mice were analyzed on the 14th day, and seven cell clusters were obtained through dimensionality reduction clustering (Figure 4A). Specific macrophage subpopulations Cluster 0 expressed Arg1 and Cd36 (Arg1hi macrophages), Cluster 1 expressed Ly6c2 and Plac8 (monocytoid macrophages), and Cluster 2 expressed Folr2 and Mrc1 (Mrc1hi macrophages). Cluster 3 expressed Dpt and Lum (colloid macrophages), Cluster 4 expressed Nlrp3 and Il-1β (proinflammatory macrophages), and Cluster 5 expressed Chchd2 and Vamp3. Cluster 6 expressed Ccl5 and Cxcl10 (Figure 4B). Among them, the proportion of specific macrophage subpopulations Cluster 0 macrophages was the greatest, indicating that cluster 0 macrophages were dominant in the local macrophages of the tissue in the later stage of wound healing (day 14). The percentages of the seven clusters in the tissue cells of the two groups were analyzed. The results revealed that the proportions of specific macrophage subpopulations Cluster 0 and Cluster 2 bacteria in the HAMA group were significantly greater than those in the control group (Figure 4C). The DEGs of macrophages derived from wounds in the two groups of mice were analyzed. The volcano plot results revealed that the expression of genes related to promoting healing, such as Arg1 and Il-10, in the skin tissue of the HAMA group was significantly greater than that in the control group, which was consistent with the phenomenon that the wounds in the HAMA group had healed, whereas those in the control group had not (Figure 4D). The GO analysis results also revealed that the two groups presented the greatest difference in "regulation of the inflammatory response" (Figure 4E).

**Figure 5.**
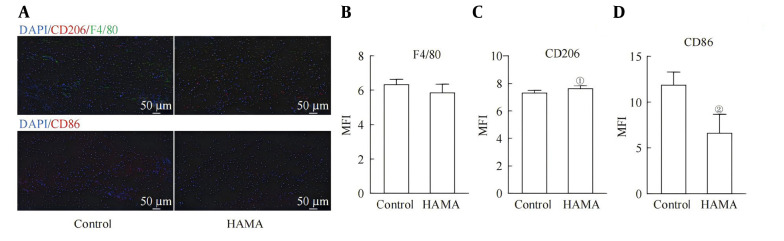
Effect of the HAMA hydrogel on the expression of polarization-related genes in macrophages

Furthermore, the mouse macrophage line Raw264.7 was coincubated with the HAMA hydrogel in vitro. The HAMA hydrogel induced the expression of the anti inflammatory related gene Arg1 in macrophages, while the expression of the proinflammatory-related gene Nos2 was significantly reduced (Figure 5).

### 3.5. Anti-inflammatory Characteristics of Macrophages in Wound Healing Tissues

**Figure 6.**
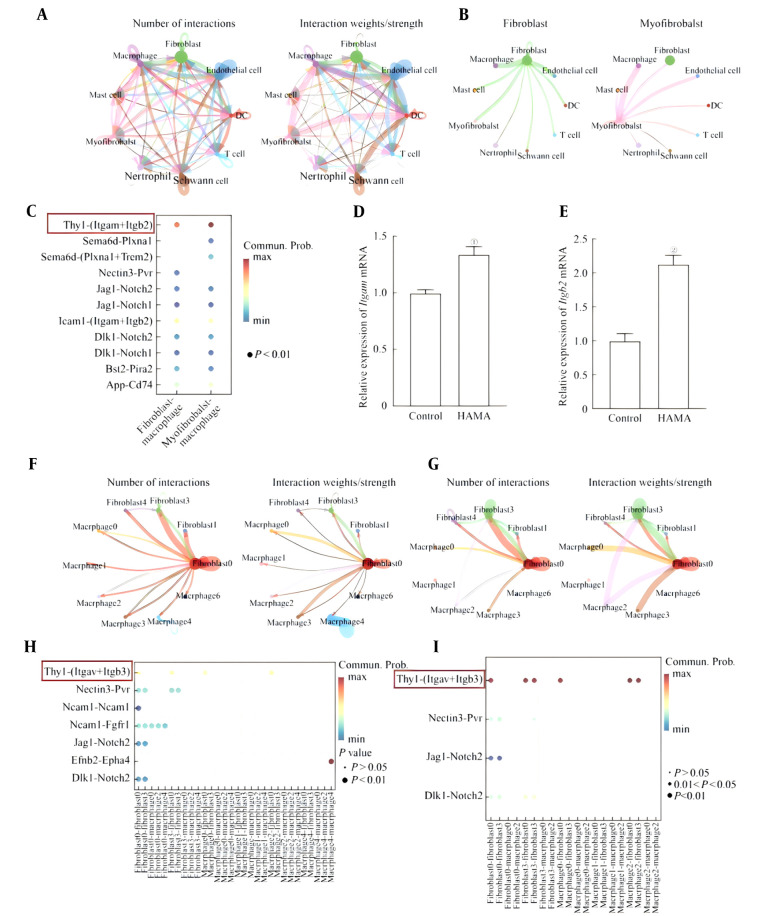
A, The number and strength of interactions among different cell types in the wound tissue (larger circles represent a greater number of cells, and thicker lines indicate stronger interaction strength); B, interactions between fibroblasts and myofibroblasts with macrophages; C, receptor-ligand pairs for interactions between fibroblasts and myofibroblasts with macrophages. D/E. mRNA expression levels of D, Itgam and E, Itgb2 in Raw264.7 cells after 24 h of incubation with solidified HAMA hydrogel or control treatment. F/G. The number and strength of interactions among various subgroups in the wound sites from the F, control group and G, HAMA group. H/I. Receptor-ligand pairs involved in interactions among various subgroups in wound sites from the H, control group and I, HAMA group. ①P < 0.001, compared with the control group.

Immunofluorescence staining was performed on macrophages at the wound healing sites of the mice (Figure 6A). Although there was no significant difference in the number of local macrophages at the wound sites between the two groups (Figure 6B), compared with the unhealed wound sites of the control group, the HAMA group presented more CD206 and less CD86 at the wound healing site (Figure 6C and D), further confirming that the macrophages at the wound healing site presented stronger anti-inflammatory characteristics after the action of the HAMA hydrogel (Figure 6).

A. Immunofluorescence of macrophages at the wound site in mice (n = 6). B-D. Mean fluorescence intensity of F4/80 (B), CD206 (C) and CD86 (D) macrophages at the wound site. ①P < 0.001, compared with the control group.

## 4. Discussion

In this study, a HAMA hydrogel was synthesized by modifying HA, and the HAMA hydrogel accelerated the healing of skin wounds in mice (23). Through single-cell sequencing of wound tissues, the characteristic differences and interaction mechanisms of macrophages and fibroblasts that play important roles in wound healing were revealed, providing an immunological basis for studying the mechanism by which HAMA hydrogel dressings promote wound healing (24- 26).

Compared with the untreated group, the control group that used LAP combined with UV irradiation did not exhibit significant differences in wound healing rate, inflammatory response, and tissue regeneration. This indicates that LAP, as a photoinitiator, has no obvious biological impact or interference on the wound microenvironment under the experimental conditions. Therefore, our choice of LAP + UV irradiation as the main control group was to control the variable effects that might be brought about by the photopolymerization process itself, in order to more accurately evaluate the specific biological functions of HAMA hydrogel. This control group design can effectively distinguish the pro-healing effect of the HAMA material itself from the influence of the photopolymerization process, ensuring the reliability of the research conclusion.

At present, hydrogels have become one of the most promising types of wound dressings (27- 29). The ideal wound dressing should retain moisture at the wound site, remove exudate from the wound, and possess basic properties similar to those of natural tissues. Electrospinning modified with mesenchymal stem cell membranes can accelerate wound repair during diabetic inflammation (30). Collagen-based hydrogels can promote angiogenesis and accelerate wound healing. HA is an important component that naturally exists in human skin and connective tissue and has good biocompatibility. This means that when the HAMA hydrogel is applied for wound healing, it causes fewer immune rejection reactions, thereby reducing the risk of complications (31).

Moreover, the HAMA hydrogel has better moisturizing properties and can form a protective film on the surface of the wound, effectively preventing water evaporation and helping to maintain cell vitality (32). Compared with biomaterials with strong mechanical support, such as nanofibers and polylactic acid, HAMA hydrogels have moderate mechanical strength and good flexibility and can adapt to movement and tension at the wound site. This study also demonstrated that HAMA hydrogels can effectively promote wound healing and are expected to become a new type of biomaterial for clinical wound treatment (33).

The results of this study demonstrate that HAMA hydrogel exhibits unique advantages compared to various skin wound repair materials reported in the literature. Compared to traditional collagen hydrogels, HAMA has superior mechanical properties and a more controllable degradation rate, providing more durable physical support during wound healing. Compared to chitosan-based materials, HAMA has better biocompatibility and lower immunogenicity, reducing the risk of inflammation. Compared to synthetic polymers such as polylactic acid, HAMA, as a natural polysaccharide derivative, has better cell affinity and biological activity, effectively promoting cell migration and proliferation. The photopolymerization property of HAMA hydrogel enables in-situ gelation, making clinical application more convenient. Additionally, its excellent moisturizing properties provide an ideal microenvironment for wound healing. Moreover, this study confirms that HAMA can effectively regulate macrophage polarization, promoting the differentiation of M2-type macrophages, thereby accelerating inflammation resolution and tissue regeneration. This advantage is not possessed by many traditional materials.

After skin injury, fibroblasts can express damage-related molecular patterns, stress response genes, proinflammatory cytokines and chemokines and participate in the inflammatory response. In tissue repair and regeneration, fibroblasts play a core role and are promoted by generating extracellular matrix and matrix molecules. Fibroblasts are the main components of connective tissue and are responsible for synthesizing and remodeling the extracellular matrix, providing structural support for tissue repair (34). Fibroblasts secrete growth factors such as TGF-β and platelet-derived growth factor. PDGF and vascular endothelial growth factor (VEGF) promote the proliferation and migration of cells at the wound site, form granulation tissue, and promote the formation of new blood vessels. It provides essential nutrients and oxygen for the regeneration of tissues (35). Different types of fibroblasts play different roles in the healing process, and type III collagen can promote the healing of skin wounds. In this study, through single-cell transcriptomic analysis of mouse wound tissues and fluorescence staining of immunotissues, we found that in the later stage of wound healing (day 14), the HAMA group had more fibroblasts that secreted type III collagen that converged locally at the wound site, which was closely related to accelerated wound healing (36). However, the subpopulation differentiation mechanism of local fibroblasts on the wound surface after HAMA treatment remains unclear. HAMA can induce macrophages to polarize toward the phenotype of anti-inflammatory macrophages, whereas activated macrophages stimulate fibroblast proliferation and collagen synthesis (37). The physicochemical properties of the HAMA hydrogel make it possible to activate fibroblasts through mechanically sensitive pathways (38). HAMA hydrogels can trigger fibroblast activation through receptors, and the newly formed collagen further forms positive feedback, promoting extracellular matrix remodeling (39). This study utilized single-cell sequencing to obtain fibroblast subpopulations capable of secreting different types of collagen, providing direct evidence for a deeper understanding of the functional segmentation of fibroblasts during wound healing. Fibroblasts are closely related to immune cells, especially macrophages. In the inflammatory response, fibroblasts and macrophages work together to form an anti-inflammatory environment. Studies have reported that fibroblasts secrete fibroblast growth factor, cytokines and chemokines to regulate the polarization of macrophages, influencing their transformation from a proinflammatory state to an anti-inflammatory state (40). During the process of wound repair, fibroblasts are also involved in regulating the survival and death signals of proinflammatory macrophage subsets (41). In this study, the communication between anti-inflammatory macrophages and fibroblasts expressing type III collagen in the wound tissue derived from the HAMA hydrogel was significantly enhanced, which is consistent with its ability to accelerate wound healing. These findings further confirmed that specific functional subgroups of fibroblasts and macrophages promote wound healing in a synergistic manner.

The HAMA hydrogel possesses excellent moisturizing properties, creating a moist environment for wounds and accelerating the migration and proliferation of epithelial cells. Additionally, as a derivative of hyaluronic acid, HAMA can specifically bind to the CD44 receptor, activate downstream signaling pathways, and promote fibroblast activation and collagen synthesis. This study has found that HAMA can regulate macrophage polarization, promote the differentiation of M2-type macrophages, accelerate inflammation resolution and tissue repair. Moreover, the hyaluronic acid fragments released during the degradation of H water gel have biological activity, which can stimulate the proliferation of vascular endothelial cells and promote the formation of new blood vessels. The three-dimensional network structure of HAMA provides an ideal growth scaffold for cells, simulates the extracellular microenvironment, and guides the orderly regeneration of tissues. By regulating the inflammatory response, promoting angiogenesis and tissue regeneration, the HAMA hydrogel achieves comprehensive regulation of the wound healing process, demonstrating a more superior effect in promoting wound healing compared to traditional materials.

## 5. Conclusion

HAMA hydrogels can promote wound healing and alter the local immune-matrix microenvironment of the wound, including increasing the proportion of anti-inflammatory macrophages, increasing type III collagen, and altering the interaction between macrophages and specific fibroblast subsets, thereby enhancing the tissue repair functions of fibroblasts and macrophages and promoting skin wound healing.

## Data Availability

The data presented in this study are uploaded during submission as a supplementary file and are openly available for readers upon request.
